# The Gametocytocidal Efficacy of Different Single Doses of Primaquine with Dihydroartemisinin-piperaquine in Asymptomatic Parasite Carriers in The Gambia: A Randomized Controlled Trial^☆^

**DOI:** 10.1016/j.ebiom.2016.10.032

**Published:** 2016-10-23

**Authors:** Joseph Okebe, Teun Bousema, Muna Affara, Gian Luca Di Tanna, Edgard Dabira, Abdoulaye Gaye, Frank Sanya-Isijola, Henry Badji, Simon Correa, Davis Nwakanma, Jean-Pierre Van Geertruyden, Chris Drakeley, Umberto D'Alessandro

**Affiliations:** a Disease Control & Elimination Theme, Medical Research Council Unit, Fajara, The Gambia; b Department of Immunology and Infection, Faculty of Infectious and Tropical Diseases, London School of Hygiene and Tropical Medicine, London, United Kingdom; c Department of Medical Microbiology, Radboud University Medical Center, Nijmegen, The Netherlands; d Pragmatic Clinical Trials Unit, Centre for Primary Care and Public Health, Queen Mary University of London, United Kingdom; e Epidemiology for Global Health Institute, Faculty of Medicine & Health Sciences, University of Antwerp, Antwerp, Belgium; f Department of Disease Control, Faculty of Infectious and Tropical Diseases, London School of Hygiene and Tropical Medicine, London, United Kingdom; g Department of Public Health, Institute of Tropical Medicine, Antwerp, Belgium

**Keywords:** ACT, artemisinin-based combination therapy, ANOVA, analysis of variance, CI, confidence interval, CYP2D6, cytochrome P450 2D6, DHAP, dihydroartemisinin-piperaquine, G6PD, glucose-6-phosphate dehydrogenase, HR, hazards ratio, IQR, interquartile ratio, MD, mean difference, MRC, Medical Research Council, SD, standard deviation, PQ, primaquine, QT-NASBA, quantitative nucleic acid sequence based assay, Asymptomatic infection, Malaria, Primaquine, Plasmodium falciparum, Infectivity, Gametocyte carriage, Efficacy, Randomized trial

## Abstract

**Background:**

Asymptomatic low-density gametocyte carriers represent the majority of malaria-infected individuals. However, the impact of recommended treatment with single low dose of primaquine and an artemisinin-based combination therapy to reduce transmission in this group is unknown.

**Methods:**

This was a four-arm, open label, randomized controlled trial comparing the effect of dihydroartemisinin-piperaquine (DHAP) alone or combined with single dose of primaquine (PQ) at 0.20 mg/kg, 0.40 mg/kg, or 0.75 mg/kg on *Plasmodium falciparum* gametocytaemia, infectiousness to mosquitoes and hemoglobin change in asymptomatic, malaria-infected, glucose-6-phosphate dehydrogenase (G6PD) normal individuals. Randomization was done using a computer-generated sequence of uneven block sizes with codes concealed in sequentially numbered opaque envelopes. The primary endpoint was the prevalence of *P*. *falciparum* gametocytemia at day 7 of follow-up determined by quantitative nucleic acid sequence based assay and analysis was by intention to treat. The trial has been concluded (registration number: NCT01838902; https://clinicaltrials.gov/ct2/show/NCT01838902).

**Results:**

A total of 694 asymptomatic, malaria-infected individuals were enrolled. Gametocyte prevalence at day 7 was 37.0% (54/146; 95% CI 29.2–45.4), 19.0% (27/142; 95% CI 12.9–26.4), 17.2% (25/145; 95% CI 11.0–23.5) and 10.6% (15/141; 95% CI 6.1–16.9) in the DHAP alone, 0.20 mg/kg, 0.40 mg/kg, and 0.75 mg/kg PQ arms, respectively. The main adverse events reported include headache (130/471, 27.6%), cough (73/471, 15.5%), history of fever (61/471, 13.0%) and abdominal pain (57/471, 12.1%). There were five serious adverse events however, none was related to the interventions.

**Interpretation:**

A single course of PQ significantly reduces gametocyte carriage in malaria-infected asymptomatic, G6PD-normal individuals without increasing the risk of clinical anemia. The limited number of successful mosquito infections suggests that post-treatment transmission potential in this asymptomatic population is low.

## Highlights

• Treatment with primaquine and an artemisinin-based combination therapy is part of malaria elimination strategies.
• Its slow uptake, despite specific recommendations highlights the need for evidence on primaquine's efficacy and safety.
• About 50% of asymptomatic *P*. *falciparum*-infected individuals carry gametocytes detectable by sensitive molecular methods.
• Primaquine reduced gametocyte prevalence at day 7 and carriage time in the study population at all doses tested.

Adding a single dose (0.75 mg/kg) of primaquine to the treatment of uncomplicated falciparum malaria was recommended in 2010 on the basis of weak evidence. Primaquine, at doses as low as 0.20 mg/kg, can reduce substantially gametocyte carriage in asymptomatic, malaria-infected individuals, representing the majority of the human reservoir of infection. Post-treatment transmission potential is low with artemisinin-based combination alone or with the 0.20 mg/kg dose but may occur despite reductions in gametocyte carriage.

## 1 Introduction

Asymptomatic, low-density infections constitute over 60% of the human reservoir of malaria parasite (Laishram et al., 2012) and this combined with long periods of carriage without progression to clinical disease (Bereczky et al., 2004), even in low transmission settings, suggests that asymptomatically infected individuals may contribute substantially to malaria transmission (Alves et al., 2005, Mwesigwa et al., 2015). In contrast, clinical malaria cases have been associated with higher parasite densities. However, the relation between pre-treatment asexual parasite density and gametocyte prevalence after treatment has not been consistent. Antimalarial treatment clears the asexual parasite load which in turn reduces gametocyte burden but clearance of mature gametocytes present prior to treatment is incomplete and varies by treatment (WWARN Gametocyte Study Group, 2016). Therefore, for interrupting malaria transmission and eventual elimination, efficient surveillance and treatment of all persons infected with both asexual stages and gametocytes (Slater et al., 2015) is important.

Primaquine (PQ), an 8-aminoquinoline, is recommended in combination with an artemisinin-based combination therapy (ACT) in low *Plasmodium falciparum* transmission settings to further reduce transmission (World Health Organization, 2010). These drugs act complimentarily: ACTs rapidly clear the *P*. *falciparum* asexual parasite biomass as well as early gametocyte stages (Chotivanich et al., 2006), considerably reducing post-treatment gametocyte carriage (WWARN Gametocyte Study Group, 2016) while PQ clears mature gametocytes (White, 2008). However, implementation has been slow because PQ causes a dose-dependent hemolysis, particularly in individuals with some deficiency of the red blood cell enzyme, glucose 6-phosphate dehydrogenase (G6PD) (Eziefula et al., 2014b). The mean prevalence of G6PD deficiency variant in sub-Saharan Africa is 7.5% (Nkhoma et al., 2009) but varies significantly by and within country (Howes et al., 2012). Lower PQ doses may reduce the risk of hemolytic events. The recommended dose was recently reduced from 0·75 mg base/kg to 0·25 mg base/kg to minimize this risk of hemolysis (Global Malaria Programme, 2015) while presumably retaining efficacy (Ashley et al., 2014, WHITE et al., 2012). PQ's mode of action is unclear but may act by sterilizing gametocytes and thus preventing fertilization in the mosquito; this effect precedes clearance of gametocytes from circulation (White, 2013). The presence of circulating gametocytes is thus a poor predictor of transmissibility (Karunajeewa and Mueller, 2016). The efficacy of PQ has been measured by gametocyte clearance and infectiousness to mosquitoes. However, infectiousness studies are not well standardized and this affects their suitability for evaluating efficacy of transmission-blocking interventions (Bousema et al., 2012).

Early trials on the 0.75 mg/kg dose reported variable reductions in gametocyte carriage (El-Sayed et al., 2007, Shekalaghe et al., 2011, Von Seidlein et al., 2003) and a dose-ranging trial in clinically ill patients showed efficacy at doses as low as 0.40 mg/kg (Eziefula et al., 2014a); however, non-inferiority of the 0·1 mg/kg dose was inconclusive (Eziefula et al., 2014a). More recent studies in high transmission areas and high-density gametocyte carriage confirm that PQ reduces gametocyte carriage and infectiousness to mosquitoes over a range of doses that includes the currently recommended 0.25 mg/kg (Dicko et al., 2016, Goncalves et al., 2016). There are indications that the added value of PQ in reducing post-treatment infectivity may differ between symptomatic and asymptomatic infections (El-Sayed et al., 2007, Shekalaghe et al., 2007) and the dynamics of asymptomatic low-density parasite carriage may be markedly different (WWARN Gametocyte Study Group, 2016), perhaps due to longer periods of infection in asymptomatic infections and hence more circulating mature gametocytes (Price et al., 1999, Stepniewska et al., 2008). Therefore, there is the need for evidence on the efficacy and safety of these PQ doses in asymptomatic individuals with low-density malaria infections and their impact on infectiousness to mosquitoes.

This study compared the gametocytocidal efficacy of three different single doses of PQ combined with dihydroartemisinin-piperaquine (DHAP) on gametocyte carriage in asymptomatic, malaria-infected, G6PD-normal individuals in The Gambia. Infectivity to *Anopheles coluzzii* mosquitoes was measured in a subset of enrolled participants.

## 2 Method

### 2.1 Study Design and Participants

This was a 4-arm, parallel open-label randomized controlled trial conducted in the Central and Upper River Regions of The Gambia. The trial protocol with details of the design has been published previously (Okebe et al., 2015). Asymptomatic individuals with *P*. *falciparum* mono-infection and a parasite density > 20 parasites/μl were identified by systematic community-based screening after an informed consent. Additional eligibility criteria included: age > 1 year, axillary temperature < 37.5 °C and no history of fever in the 24 h before the visit, normal G6PD status determined by fluorescent spot test (N. Dimopoulos SA) and a hemoglobin value ≥ 8 g/dl (HemoCue, Ängelholm, Sweden). Individuals who reported sickle cell disease, antimalarial use within two weeks prior to screening, current pregnancy or history of allergy to the study drugs were excluded.

### 2.2 Randomization and Allocation Concealment

All participants received DHAP (Sigma-Tau IFR S.p.A, Italy) and in addition, three of the four arms were randomized to receive a single dose of PQ at 0.75 mg, 0.40 mg or 0.20 mg base/kg body weight using a computer-generated sequence of uneven block sizes. In the first phase of the study (participants 1–347) PQ was procured from the Government Pharmaceutical Organization in Thailand; during the second phase of the study (participants 348–694) PQ was procured from Sanofi®, (Forest Park, GA, USA). The source was changed to harmonize the intervention with other on-going trials with PQ (Eziefula et al., 2012). The randomization codes for the trial arms were concealed in sequentially numbered opaque envelopes and opened by the study clinician to reveal the allocation arm and, based on body weight, calculate the dose for both DHAP and PQ. A separate randomization sequence was used to select a third of those enrolled in the trial for membrane feeding experiments.

### 2.3 Treatment and Procedures

DHAP was given once daily for 3 days on an empty stomach while PQ was given on the last day of ACT treatment at least one hour after the last dose of DHAP with a light snack. For small children, a tablet of PQ (15 mg base) was crushed and reconstituted in 15 ml of water to produce a 1 mg/ml suspension and the required dose was drawn, to the nearest 0.1 ml, mixed with orange juice and administered. A study nurse directly supervised all treatments and the participant was observed for 30 min for any reaction; if the drug was vomited within this period, it was repeated.

The choice of administration of PQ on day 2 was based on available evidence (Eziefula et al., 2012). ACTs have been shown to sharply reduce gametocyte density with the most pronounced effect within the first three days of administration, after which there is a more gradual decay of persisting gametocytes (Bousema et al., 2010). Therefore, giving PQ on day 2 provides a clearer indication of its gametocyte-clearing effect. Also, administering PQ on day 2 could limit the impact of both the clinical disease and treatment on hemoglobin levels (Eziefula et al., 2014a).

Participants were reviewed on days 1, 2, 3, 7, 10, 14, and then weekly till day 42 with clinical examinations and blood samples collected for hemoglobin check and for quantifying gametocytes marker *Pfs*25 mRNA by quantitative nucleic acid sequence based amplification (QT-NASBA) according to published protocols (Schneider et al., 2004). For direct membrane feeding experiments, 3 ml of venous blood was collected on day 7 and fed to 100 locally reared 4–5 day old female *Anopheles coluzzii* mosquitoes through an artificial membrane (Ouédraogo et al., 2013). Unfed and partially fed mosquitoes were removed after feeding while fully fed mosquitoes were kept at 28 (± 1) °C and 75% humidity for seven days. Surviving mosquitoes were dissected in 1% mercurochrome (Valdafrique Laboratoires Canonne, Rufisque) and their midguts examined for oocysts under the microscope.

Giemsa stained blood slides were examined under a microscope for asexual stage parasites and when seen, were counted against 500 white blood cells (assuming white blood cells count of 8000/μl). A slide was declared negative if no parasites were seen after examining 100 high-power fields. Microscopy results were primarily used for the clinical care of participants.

## 3 Data Management

Clinical data were single-entered onto the database (OpenClinica LLC, Waltham, MA, USA) and verified from the source documents while DMFA results were double entered on a Microsoft Access database (Microsoft Corp., Richmond, WA, USA). The QT-NASBA results were automatically extracted as Microsoft Excel document (Microsoft Corp., Richmond, WA, USA) and linked to the rest of the dataset.

### 3.1 Outcomes

The primary study endpoint was the prevalence of *P*. *falciparum* gametocytemia at day 7 of follow-up determined by QT-NASBA. Secondary outcomes were gametocyte prevalence during follow up, change in hemoglobin values during follow-up, infectiousness to mosquitoes on day 7 defined by the number of individuals with feeds resulting in ≥ 1 infected mosquito, the number of adverse and serious adverse events. The day 7 primary endpoint takes into account PQ's relatively short half-life and the period of maximum effect demonstrated in previous studies (Eziefula et al., 2014a, Shekalaghe et al., 2007), and it is in line with the standardized endpoints proposed by the Primaquine in Africa Discussion Group (Eziefula et al., 2012).

### 3.2 Sample Size Calculations

The study set out to test two hypotheses: i) PQ, at any dose, added to a full course of DHAP, results in lower gametocyte carriage compared to DHAP alone and ii) both the 0.40 mg and 0.20 mg base/kg PQ doses have similar gametocytocidal efficacies compared to the 0.75 mg base/kg. We assumed gametocyte prevalence on day 7 of 25% for the ACT alone arm in low transmission settings (Slater et al., 2015) and a 50% reduction with PQ added, at any dose. A sample size of 220/arm had 90% power and 5% significance to detect this difference in prevalence, allowing for 10% non-compliance or loss to follow-up. For the second hypothesis of non-inferiority of lower PQ doses compared to 0.75 mg base/kg, 300 participants/arm would have 90% power to detect that a confidence interval around the prevalence in the lower dose PQ arm was 8% less in the 0.2 or 0.4 mg/kg PQ arms, assuming the prevalence of 13% at day 7 in the 0.75 mg base/kg arm.

## 4 Statistical Analysis

Descriptive statistics are presented by treatment arm at baseline and gametocyte carriage compared using one way Analysis of Variance (ANOVA) on log-transformed gametocyte densities at analyzed visits. We determined the odds of gametocytemia on days 7, 10 and 14 in a multivariable logistic regression model using a generalized estimating equation with exchangeable correlation structure to control for potential confounding/effect modification due to age, gender, baseline hemoglobin level and asexual parasite count. We fitted a Cox proportional hazard model to determine the effect of PQ dose on gametocyte clearance compared to the control adjusted for baseline asexual parasite density and hemoglobin in those with gametocytemia on enrolment. For this specific analysis only, baseline gametocytemia was defined as all QT-NASBA positive samples on either day 0 or day 3 (one day post-PQ dose) to adjust for fluctuation in gametocyte densities around the threshold of detection of the QT-NASBA and hence gametocytemia as changes during this period is most likely be due to the effect of the ACT. A Kaplan Meier estimator survival plot was used to show probabilities of gametocyte carriage over time in the study arms. The change in hemoglobin levels from baseline was calculated as the difference between the reference visit and baseline. All analyses were restricted to gametocyte results between day 0 and 14 to minimize bias from possible new asexual infections that lead to *de novo* gametocyte production, as DHAP concentrations beyond this time may no longer prevent the emergence of new infections (Bousema et al., 2010). Analyses were done using Stata 13.1 (Stata Corp, College Station, TX, USA) and based on an intention-to-treat principle.

## 5 Ethics, Approvals and Trial Management

An independent Trial Steering Committee and a Data Safety and Management Board reviewed the trial conduct. The trial was approved by The Gambia Government/MRC Joint Ethics Committee (SCC 1321, 13 March 2013) and the London School of Hygiene and Tropical Medicine Research Ethics Committee (ref 6412). A signed informed consent was obtained from all participants; consent for children was provided by the caregiver and an assent obtained from children between 12 and 17 years. The trial was conducted in conformity with the principles of good clinical practice and the Helsinki Declaration.

## 6 Changes to the Trial Protocol after Initiation of the Study

The site in the Central River Region was relocated to Diabugu in the Upper River Region due to low recruitment. However, due to distance of this new site to the insectary, feeding experiments were limited to one site and the randomization sequence adjusted while maintaining group allocation.

At the end of the second recruitment season, the Trial Steering Committee reviewed the progress of the trial against the planned sample size and the funding period. Although the number of individuals recruited was lower than the target sample size, the trial was sufficiently powered to test the first trial hypothesis. However, a much larger sample, beyond the initial projection, was required to prove non-inferiority between PQ arms. Therefore, it was decided to close the trial.

## 7 Results

**Fig. 1:**
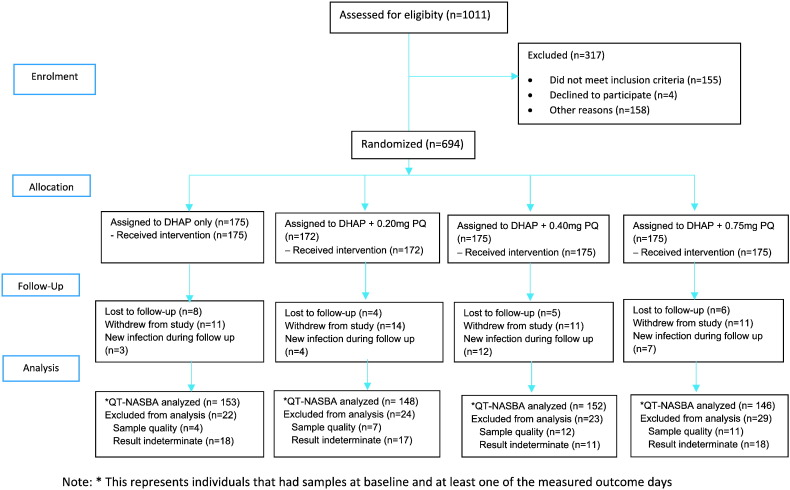
Flow diagram of the study. DHAP: dihydroartemisinin-piperaquine, PQ: primaquine, QT-NASBA: quantitative nucleic acid sequence-based assay.

The trial was conducted over two transmission seasons: August 2013–February 2014 and August 2014–February 2015. The systematic community-based screening identified 1011 persons with parasite density > 20 parasites/μl of which 694 (68.6%) were enrolled. There were 306 (44·1%) males and the median (IQR) age and weight were 10 (7–14) years and 28.2 (20.3–38.7) kg, respectively. Forty-seven (6 · 8%) participants withdrew from the study after enrolment; 27 of these withdrew before day 7, and 24 (3 · 5%) were lost to follow up (Fig. 1). The baseline gametocyte prevalence was 12 · 5% (85/680) by microscopy and 52 · 3% (313/599) by QT-NASBA, with no difference between treatment arms (Table 1). A total of 170 participants participated in membrane feeding assays.

### 7.1 Primary Endpoint: Gametocyte Carriage on Day 7 After Treatment

**Fig. 2:**
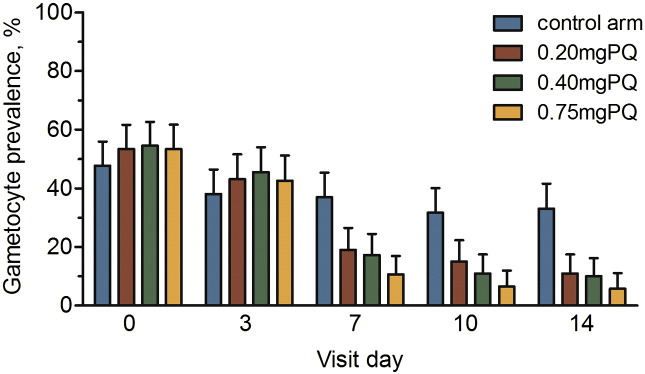
Gametocyte prevalence in each trial arms for each analyzed day of follow up. Error bars indicates the upper limit of the 95% confidence interval around the prevalence.

**Fig. 3:**
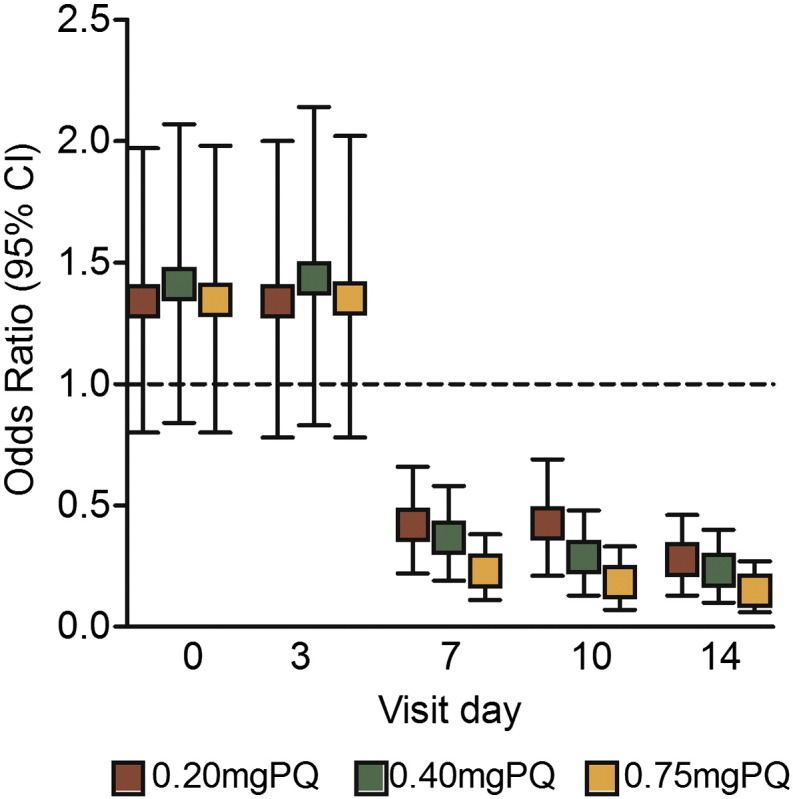
Odds ratio of gametocyte prevalence on each of the days of follow-up compared with the control arm adjusted for baseline gametocyte density. Bars indicate the upper and lower limits of the 95% confidence.

The drop in gametocyte prevalence between day 0 and day 7 was significantly lower in all PQ arms compared to the DHAP alone arm, with a mean difference (MD) of 34.3% (95% CI 24.1–44.7), 37.4% (95% CI 27.3–47.4) and 42.8% (95% CI 33.2–52.3) in the 0.20 mg, 0.40 mg and 0.75 mg PQ arms, respectively (Fig. 2). A chi squared test showed a significant trend in gametocyte reduction on day 7 between study arms (p < 0.001). Participants who received PQ arms had lower odds of gametocytemia at day 7 compared to the DHAP arm: 0.20 mg (OR 0·38; 95% CI 0.22–0.66; p < 0.001), 0.40 mg (OR 0.33; 95% CI 0.19–0.58; p < 0.001), and 0.75 mg (OR 0.20; 95% CI 0.11–0.38; p < 0.001), after adjustment for baseline gametocyte density (Fig. 3).

### 7.2 Secondary Endpoint: Gametocyte Carriage During Follow Up

Gametocyte prevalence on day 3, one-day post PQ administration, was not significantly different compared to baseline in the control (MD 9.6%; 95% CI–1.5 to 20.7; p = 0.092), 0.20 mg PQ (MD 10.2%; 95% CI–1.1 to 21.6; p = 0.079), 0.40 mg PQ (MD 9.0%; 95% CI–2.2 to 20.3; p = 0.119) and 0.75 mg PQ (MD 10.8%; 95% CI–0.6 to 22.2; p = 0.067) arms.

**Fig. 4:**
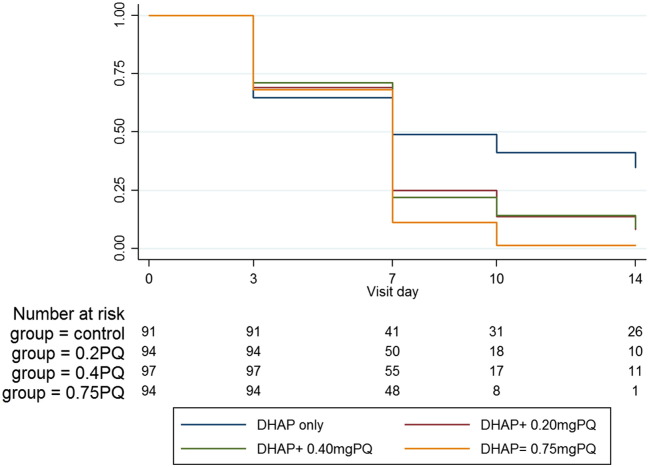
Kaplan Meier plot for gametocyte clearance in individuals who were gametocyte positive at enrolment.

By day 14, prevalence was significantly lower from baseline in the 0.20 mg (MD 42.4%; 95% CI 32.8–52.1; p < 0.001), 0.40 mg (MD 44.6%; 95% CI 35.3–54.0; p < 0.001) and the 0.75 mg PQ arms (MD 47.6%; 95% CI 38.4–56.6; p < 0.001) than in the control (MD 14.6%; 95% CI 3.5–25.7; p = 0.011). The odds of gametocytemia remained significantly lower in the 0.20 mg (OR 0.24; 95% CI 0.13–0.46; p < 0.001), 0.40 mg (OR 0.20; 95% CI 0.10–0.40; p < 0.001) and the 0.75 mg/kg (OR 0.12; 95% CI 0.06–0.27; p < 0.001) PQ arms after adjusting for baseline gametocyte density. Although the odds of gametocytemia was lowest in the 0.75 mg arm, there was no significant difference between the PQ arms (Fig. 3). Gametocyte clearance was associated with PQ dose, being fastest in the 0.75 mg PQ arm (hazard ratio (HR) 1.81; 95% CI 1.25–2.62; p = 0.002) followed by the 0.40 mg PQ (HR 1.53; 95% CI 1.06–2.20; p = 0.023) and the 0.20 mg PQ arm (HR 1.49; 95% CI 1·03–2·16; p = 0·033) after adjusting for asexual parasite density and hemoglobin level (Fig. 4).

### 7.3 Secondary Endpoint: Infectivity to Mosquitoes in Membrane Feeding Assays

The prevalence of gametocytemia at day 7, by microscopy and QT-NASBA was 2 · 5% (4/161) and 28 · 0% (45/161), respectively (Table 2). Two participants; in the control and the 0·20 mg/kg PQ arms each infected ≥ 1 mosquito. In the control arm, one mosquito was infected (1 · 3%, 1/80) with two oocysts while in the 0·20 mg/kg PQ arm, 29 · 2% (19/65) of fed mosquitoes were infected, with oocyst densities ranging between 1 and 3 per infected mosquito (27 in total). Both individuals had gametocytes detected by QT-NASBA on day 7; the participant in the control arm also had gametocytes detected by microscopy.

### 7.4 Secondary Endpoint: Safety, Change in Hemoglobin Concentrations

The mean (SD) hemoglobin at baseline was 11 · 5 (1 · 6) g/dl and did not vary significantly between treatment arms (Table 1). Fifty-three percent (337/637) of study subjects experienced a drop in hemoglobin at day 7. However, 97.3% (328/337) of these were ≤ 25% of the baseline value. The mean change in hemoglobin was largest on day 3; however, most participants had returned to baseline values by day 10. Overall, the mean (95% CI) change in hemoglobin throughout the follow up was 0.12 g/dl (− 0.01 to 0.25), 0.26 g/dl (0.14–0.38), 0.41 g/dl (0.29–0.53) and 0.19 g/dl (0.05–0.33) in the control, 0.20 mg, 0.40 mg and 0.75 mg PQ arms, respectively. The lowest hemoglobin value documented in the trial was 4.7 g/dl recorded on day 3 in a 7-year old boy in the 0.75 mg PQ arm (starting value 8.3 g/dl) while the maximal fall in hemoglobin value during follow-up was 7.5 g/dl on day 7 (starting value 14.3 g/dl) in a female in the control arm. These participants did not show clinical signs but were treated with hematinics as per protocol and recovered to baseline levels during follow up. Overall, no clinical symptoms were observed with the changes in hemoglobin.

### 7.5 Secondary Endpoint: Adverse Events

There were 452 adverse events from 471 participants observed during follow up, with no difference between treatment arms (Table 3). The main complaints were headache (130/471, 27.6%), cough (73/471, 15.5%), history of fever (61/471, 13.0%) and abdominal pain (57/471, 12.1%). One participant had to be re-dosed after vomiting DHAP. Five serious adverse events occurred during the trial and none was related to the intervention. They include clinically diagnosed pneumonia (two cases), meningitis (no organisms seen on bacterial culture), road accident and an iatrogenic abscess after receiving intramuscular quinine by a third party. All events resolved completely.

## 8 Discussion

The vast majority of malaria infections in low endemic settings are of low density (Imwong et al., 2016) but can contribute considerably to onward malaria transmission (Slater et al., 2015). However, the impact of current ACT and ACT-PQ on preventing onward malaria transmission from these low-density infections has never been established. Our study is the first to determine the effect of single low dose PQ on gametocytemia and infectivity to mosquitoes after treatment with a currently available ACT in asymptomatic, low-density infections. PQ is considered a potent gametocytocide to support community campaigns with ACT. Although high community coverage with efficacious antimalarials is arguably the most important factor in determining the success of community treatment campaigns (Johnston et al., 2014, Okell et al., 2011), the drug itself is important (Okell et al., 2011) because antimalarials differ in their prophylactic and gametocytocidal effects (Stasinopoulos and Rigby, 2007). The justification for including PQ in community treatment campaigns is that it shortens the infectious period after ACT (Dicko et al., 2016) and may thus reduce community-wide malaria transmission potential shortly after treatment. Previous studies with sulfadoxine-pyrimethamine alone or with PQ at 0.75 mg/kg concluded that PQ considerably reduced the duration of gametocyte carriage in symptomatic patients (Shekalaghe et al., 2007); however, this was not the case in asymptomatic, low-density infections (El-Sayed et al., 2007). Our study demonstrates a statistically significant reduction in the prevalence of gametocytemia and shorter gametocyte carriage time following all PQ doses compared to DHAP alone. This is consistent with the growing body of evidence showing that PQ is efficacious for reducing gametocyte carriage (Eziefula et al., 2014a, Dicko et al., 2016, Smithuis et al., 2010, Goncalves et al., 2016, Shekalaghe et al., 2007). It is currently impossible to predict infectivity to mosquitoes from molecular gametocyte markers only (White et al., 2014) and mosquito-feeding experiments remain important to draw conclusions on infectivity. Acknowledging these limitations of gametocytemia as outcome measure, we aimed to support our findings with mosquito feeding assays. We observed very low infectivity in membrane feeding experiments conducted at day 7, with one successful feed in the control arm and one in the 0.2 mg/kg PQ arm. Two previous studies that determined transmission after DHAP found that 36% (29/80) of symptomatic children (Rigby and Stasinopoulos, 2004) and 23% (3/13) of individuals with patent gametocytemia prior to treatment (Dicko et al., 2016) infected at least one mosquito on day 7 after treatment. Because of the limited number of infectious individuals in the DHAP arm, our findings do not allow conclusive statements on the transmission-blocking potential of PQ in this population. Other studies that assessed infectivity after PQ maximized the discriminative power of the feeding assays by purposefully selecting high-density gametocyte carriers, likely to be infectious before treatment (Dicko et al., 2016, Goncalves et al., 2016). We complement these findings with experiments in a population that is much less likely to be infectious but considerably more representative of the average malaria-infected population (Slater et al., 2015, Imwong et al., 2016). We performed feeding experiments on day 7 only. Two recent studies determined infectivity before and at different time-points after treatment (Dicko et al., 2016, Goncalves et al., 2016), thereby providing a valuable quality control of the sensitivity of feeding experiments, which may vary considerably between settings and mosquito sources (Smith et al., 2000). Because we only measured malaria transmission seven days post treatment, we cannot rule out a suboptimal sensitivity of our feeding assays and should therefore be cautious when interpreting the results. Our data suggest low infectivity of this population and that malaria transmission was not fully prevented in the DHAP arm and the arm with the lowest PQ dose (0.20 mg/kg).

A clinical trial on *P*. *vivax* treatment that was conducted after the initiation of the current study indicated that PQ efficacy in clearing *P*. *vivax* hypnozoites is influenced by genetic polymorphisms affecting PQ metabolism by cytochrome *P450 2D6* (CYP2D6) (Bennett et al., 2013). The relevance of CYP2D6 variants for gametocyte clearance remains to be established; we did not perform genotyping for CYP2D6 which requires larger sample volumes (Dicko et al., 2016) and therefore cannot determine its impact on gametocyte clearance or malaria transmission after PQ.

In our study population, PQ was well tolerated with no statistically significant or clinically relevant hemolysis. Treatment-associated hemolysis has been previously observed following a single dose PQ at 0.75 mg/kg (Eziefula et al., 2014a, Shekalaghe et al., 2010) and 0.4 mg/kg (Eziefula et al., 2014a) and has been associated with G6PD deficiency. In our trial, hemoglobin changes were observed in all trial arms with the greatest difference from baseline, − 7.5 g/dl observed on day 7 in a female in the DHAP arm. However, she recovered completely without clinical symptoms due to anemia. This pattern of marked drop in hemoglobin followed by full recovery is commonly observed also in patients with acute uncomplicated falciparum malaria (Ekvall et al., 2001) and the current study does not show a marked effect of PQ on hemoglobin concentrations post-treatment. It is important to acknowledge that this trial was not designed to look at safety and included only G6PD normal individuals with relatively high hemoglobin concentrations (≥ 8 g/dl). If PQ is considered for inclusion in malaria control programs, safety studies are needed with PQ at doses within the therapeutic range in G6PD deficient individuals.

In conclusion, we report that a single course of PQ when given together with DHAP rapidly and significantly reduces gametocytemia. We observed very low levels of post-treatment infectivity to mosquitoes, which did not allow a robust comparison between treatment arms. The low level of infection raises questions about the added value of PQ in preventing transmission to mosquitoes 7 days post- treatment. Future studies should address the safety of PQ in G6PD-deficient individuals.

## Declaration of Interest

UDA received grants from Joint Global Health Trial Scheme (MRC, DFID, Wellcome Trust), during the conduct of the study; other grants from Sigma Tau Industrie Farmaceutiche Riunite and Medicine for Malaria Venture were received outside the submitted work. TB was supported through a fellowship from the European Research Council (ERC-2014-StG 639776).

## Contributors

JO, TB, DN, CD, JPV and UDA were involved in the study design, writing and interpretation. JO implemented and led the study supervised by JPV and UDA. JO and GLDT analyzed the data. ED and FSI led the clinical team in data collection and participant care. MA and HB conducted the QT-NASBA experiments supported by TB and DN. SC performed and supervised the microscopy slide readings. AG conducted the membrane feeding experiments supervised by TB and CD. All authors reviewed and approved the final version of the manuscript.

## Role of the Funding Source

The funders had no role in the trial design or the preparation of the manuscript.

## Figures and Tables

**Table 1:** Characteristics of participants at enrolment.

	DHAP only (N = 175)	DHAP + 0.20 mgPQ (N = 172)	DHAP + 0.4 mgPQ (N = 175)	DHAP + 0.75mgPQ (N = 172)
Age in years	11 (8–14)	10 (7–14)	10 (7–15)	10 (7–14)
Male (%)	88 (50.3)	71 (41.3)	76 (43.4)	71 (41.3)
Axillary temperature (°C)	36.4 (0.4)	36.4 (0.4)	36.5 (0.4)	36.4 (0.4)
Hemoglobin (g/dl)	11.6 (1.9)	11.6 (1.7)	11.4 (1.4)	11.6 (1.6)
Asexual parasite density (/μl)	193 (48–688)	221 (48–648)	215 (48–672)	294 (48–1073)
Gametocyte prevalence by microscopy	7.1 (12/170)	13.5 (23/170)	15.8 (27/171)	13.6 (23/169)
Gametocyte prevalence by QT-NASBA	47.7 (73/153)	53.4 (79/148)	54.6 (83/152)	53.4 (78/146)
Gametocyte density by QT-NASBA (/μl)	11.7 (1.4–106.7)	12.9 (1.2–127.8)	11.3 (1.4–81.9)	10.7 (1.4–84.2)

Note: means and standard deviation are presented for temperature and hemoglobin; age is presented as median and interquartile range, geometric means and interquartile ranges are presented for asexual parasite and gametocyte densities. QT-NASBA: quantitative real-time nucleic acid sequence-based analysis.

**Table 2:** Profile and results for assays on transmission to *Anopheles* mosquitoes on day 7 after initiation of treatment.

	DHAP only	DHAP + primaquine		
	DHAP (N = 46)	0.20 mg (N = 44)	0.40 mg (N = 42)	0.75 mg (N = 38)
% with gametocytemia (microscopy)^a^	6.7 (3/45)	0	2.6 (1/38)	0
% with gametocytemia (QT-NASBA)	50.0 (22/44)	12.5 (5/40)	28.6(12/42)	17.1 (6/35)
Number of mosquitoes examined, median (IQR)	80 (80–96)	80 (80–100)	80 (80–100)	80 (80–100)
Proportion of infectious individuals^b^	2.2 (1/46)	2.3 (1/44)	0 (0/42)	0 (0/39)
Proportion of infected mosquitoes	1.3 (1/80)	29.2 (19/65)	–	–
Median (range) oocyst density in infected mosquitoes	2	1 (1–3)	–	–

a Standard 100 field estimate.

b Proportion of individuals infecting at least one mosquito.

**Table 3:** Counts of adverse events reported by trial arm.

Event	DHAP (N = 106)	0.2PQ (N = 99)	0.4PQ (N = 139)	0.75PQ (N = 97)	Totals
Headache	29	28	42	31	130
Cough	14	12	27	20	73
Fever	13	16	19	13	61
Abdominal pain	13	14	21	9	57
Other	5	2	3	7	28
URTI	2	9	3	6	20
Skin infection	5	3	6	4	18
Pallor	9	4	4	1	18
Anorexia	5	4	1	2	12
Vomiting	3	2	6	0	11
Trauma	5	0	3	1	9
Stooling	1	3	1	1	6
Skin rash	0	2	3	1	6
Pneumonia	1	0	0	1	2
Meningitis	1	0	0	0	1
